# Developing a questionnaire to identify perceived barriers for implementing the Dutch physical therapy COPD clinical practice guideline

**DOI:** 10.1186/1472-6963-13-159

**Published:** 2013-05-01

**Authors:** Philip J van der Wees, Cor AM Zagers, Sara E de Die, Erik JM Hendriks, Maria WG Nijhuis-van der Sanden, Rob A de Bie

**Affiliations:** 1 Scientific Institute for Quality of Healthcare, Radboud University Nijmegen Medical Centre, Nijmegen, the Netherlands; 2 Department of Epidemiology and CAPHRI School for public health and primary care, Maastricht University, Maastricht, the Netherlands; 3 Department of Health Care Policy, Harvard Medical School, Boston, MA, USA

**Keywords:** Chronic Obstructive Pulmonary Disease (COPD), Data collection tools, Guideline, Physical therapy, Pulmonary rehabilitation

## Abstract

**Background:**

Clinical practice guidelines have been developed to assist healthcare practitioners in clinical decision making. Publication of clinical practice guidelines does not automatically lead to their uptake and barrier identification has been recognized as an important step in implementation planning. This study aimed at developing a questionnaire to identify perceived barriers for implementing the Dutch COPD guideline for physical therapists and its recommended measurement instruments.

**Methods:**

An overall questionnaire, based on two existing questionnaires, was constructed to identify barriers and facilitators for implementing the COPD guideline. The construct of the questionnaire was assessed in a cross-sectional study among 246 chest physical therapists. Factor analysis was conducted to explore underlying dimensions. Psychometric properties were analyzed using Cronbach’s alpha. Barriers and facilitators were assessed using descriptive statistics.

**Results:**

Some 139 physical therapists (57%) responded. Factor analysis revealed 4-factor and 5-factor solutions with an explained variance of 36% and 39% respectively. Cronbach’s alpha of the overall questionnaire was 0.90, and varied from 0.66 to 0.92 for the different factors. Underlying domains of the 5-factor solution were characterized as: attitude towards using measurement instruments, knowledge and skills of the physical therapist, applicability of the COPD guideline, required investment of time & money, and patient characteristics. Physical therapists showed a positive attitude toward using the COPD guideline. Main barriers for implementation were required time investment and financial constraints.

**Conclusions:**

The construct of the questionnaire revealed relevant underlying domains for the identification of barriers and facilitators for implementing the COPD guideline. The questionnaire allowed for tailoring to the target group and may be used across health care professionals as basis for in-depth analysis of barriers to specific recommendations in guidelines. The results of the questionnaire alone do not provide sufficient information to inform the development of an implementation strategy. The infrastructure for developing the guideline can be used for addressing key barriers by the guideline development group, using the questionnaire as well as in-depth analysis such as focus group interviews. Further development of methods for prospective identification of barriers and consequent tailoring of implementation interventions is required.

## Background

Chronic Obstructive Pulmonary Disease (COPD) is a chronic, life-threatening lung disease and the third leading cause of death worldwide. Active smoking is the main risk factor, while other factors such as occupational factors, infections and the role of air pollution are becoming more influential [1]. In the Netherlands approximately 325,000 people are formally diagnosed with COPD and more than 6,000 people die of COPD annually [2]. Clinical practice guidelines have been developed to assist healthcare practitioners in clinical decision making, by providing recommendations about appropriate healthcare for specific circumstances [3]. In the Netherlands an evidence-based guideline for physical therapy diagnosis and treatment in patients with COPD was issued by the Royal Dutch Society for Physical Therapy (KNGF) and developed according to a rigorous procedure [4]. An important component of the guideline is the recommended use of measurement instruments to establish parameters for treatment and evaluation [5].

Publication of guidelines does not automatically lead to their uptake and change of physical therapists’ behavior based on guideline recommendations shows room for improvement [6]. Several studies reported multiple barriers for adherence of physical therapists to clinical guidelines and measurement instruments [7-9]. Multifaceted implementation strategies are more likely to result in change of professional behavior compared to educational activities [10-12], and a comprehensive implementation strategy is essential in promoting the uptake of clinical guidelines. Such an implementation strategy should be tailored to specific barriers and facilitators [13]. Therefore, assessment of specific barriers is important for implementation planning.

The focus of many interventions to enhance uptake is aimed at health care practitioners (knowledge, skills, attitude), although social factors (influence of patients, colleagues, stakeholders) and external factors (financial, organizational, regulatory influences, guideline characteristics) are also important to address when designing implementation strategies [14].

Several theories and models exist to understand behavioral change of healthcare practitioners [15-18]. These models suggest that behavior is determined by three important factors: attitude, defined as positive and negative beliefs associated with a particular behavior; social norms, defined as beliefs of reference persons about a particular behavior; and self-efficacy expectations, defined as abilities to perform a particular behavior. Such models have been used to develop frameworks for implementation in order to identify barriers to change at different healthcare levels [14]. Each type of barrier may require a specific intervention, and the analysis of facilitators and barriers is a crucial step in a systematic approach for designing implementation interventions. Methods such as intervention mapping [16] or the behavior change wheel approach [19] can be used to design and select interventions.

A recently updated systematic review showed that interventions tailored to prospectively identified barriers are more likely to improve professional practice compared to no intervention or dissemination of guidelines only. Barriers are typically identified using interviews and questionnaires, while the most effective ways to identify barriers are unknown [20]. There is a need for valid methods to identify specific barriers for the topic of interest, integrated in comprehensive approaches for designing implementation strategies.

In the Netherlands a questionnaire was developed and tested among general practitioners to identify barriers for the implementation of clinical practice guidelines [21]. The questionnaire allows for tailoring to specific context and target groups, and has been used in follow-up studies to conduct surveys among Dutch general practitioners and physical therapists [22,23]. To identify barriers towards the use of measurement instruments, a general questionnaire was developed in assessing the attitude of physical therapists by asking their beliefs about the use of measurement instruments and reasons for (not) using measurement instruments [24]. We were interested whether the combined use of these two available questionnaires may be useful to identify barriers to implementing the Dutch physical therapy COPD guideline.

The specific objective of our study was to construct a questionnaire for reliable and valid assessment of barriers and facilitators among Dutch physical therapists for implementing the COPD guideline and its recommended measurement instruments.

## Methods

### Study design

We conducted a cross-sectional survey amongst Dutch physical therapists to assess the reliability and validity of a questionnaire to identify barriers for implementing the Dutch physical therapy COPD guideline and its recommended measurement instruments. Previously developed questionnaires in the Netherlands [21,24] were used for constructing an overall questionnaire in two separate parts. The original Peters questionnaire to identify barriers for implementing innovations contained 28 items based on a list of topics, and was aimed at implementation of guidelines (n = 17) and preventive activities (n = 11). The Peters questionnaire allows for tailoring and addition of items. In assessing the psychometric properties, the original Peters questionnaire revealed four domains: guideline, health care practitioner, patient and context. Cronbach’s alpha’s for internal consistency varied from 0.63 to 0.68 for the four domains [21]. The original Pisters questionnaire to assess the attitude of physical therapists towards implementing measurement instruments consisted of 22 items, divided into three parts: attitude towards the use of measurement instruments, reasons for using measurement instruments and reasons for not using measurement instruments. The Pisters questionnaire showed a Cronbach’s alpha of 0.80 [24].

### Construction of the questionnaire

A first draft of the questionnaire was devised by CZ and reviewed by PW [25,26]. For the first part of the questionnaire, all items (n = 17) of the Peters questionnaire related to guideline implementation were used and specified for the target group [21]. Additional items for guideline implementation barriers were initially derived by two members (PW, CZ) of the research group and checked by a third researcher (EH). The items were then independently scored by three experts for relevance, and consequently approved for inclusion by the research team. We added four items from the topic list of Peters that were relevant for our target group: specificity of guideline recommendations, knowledge of physical therapists, skills of physical therapists, and required time investment for using the guideline. Since socio-economic status of patients and cultural background are considered important in patients with COPD [27], two related items were added to reflect these topics. Another item was added related to the attitude of physical therapists in adopting the COPD guideline. This resulted in 24 items related to implementation barriers and facilitators for the COPD guideline. The second part of the questionnaire was aimed at the attitude towards using measurement instruments as recommended in the COPD guideline, for which we used all 22 items of the Pisters questionnaire [24].

A 5-point Likert scale was used for response categories, derived from the Peters and Pisters questionnaires: ‘strongly agree’, ‘agree’, ‘neither agree nor disagree’, ‘disagree’, ‘strongly disagree’. To assess face validity the draft questionnaire was sent to three experts who were asked to offer suggestions for improved content, wording and flow of questions. Editorial changes were made based on consensus between CZ and PW. The outline of the two parts of the questionnaire is presented in Table 1. In Additional file 1 all items of part 1 (n = 24) and part 2 (n = 22) of the questionnaire are presented.

**Table 1:** Outline of overall questionnaire to identify barriers for implementing the COPD guideline

**Domains**	**N**
**Part 1. Barriers and facilitators for guideline implementation**	**24**
Guideline	6
Health Care Practitioner	7
Patient	3
Context	8
**Part 2. Barriers and facilitators for using measurement instruments**	**22**
Attitude towards using measurement instruments	8
Reasons for using measurement instruments	5
Reasons for not using measurement instruments	9
**Total # items**	**46**

### Participants

The target group for the study was a specific group of chest physical therapists with post-graduate training in COPD. In the Netherlands these physical therapists are organized in regional networks and seven networks participated with a total of 246 physical therapists who were all approached ensuring adequate geographic distribution (Table 2).

**Table 2:** Distribution of responses to questionnaire

**Region**	**Approached**	**Response**	
	**N**	**N**	**(%)**
Central (Utrecht)	50	39	(79)
North (Groningen)	22	10	(45)
East (Nijmegen)	44	25	(57)
South (Limburg)	30	16	(54)
South (Eindhoven)	17	9	(52)
West (Amsterdam)	45	23	(51)
West (Haarlem)	38	17	(45)
**Total**	**246**	**139**	**(57)**

### Data collection

From December 2009 to June 2010 participants in the seven regions were invited via email by the researchers with a hyperlink to the online questionnaires. Email addresses of participants were obtained via the network coordinators. The responses of the participants were collected using the Formdesk™ online form management system. Individual responses were confidentially used and known only to the researchers. The data were entered in a secured database. Reminders were sent three weeks after initial distribution. We received permission for our study from the Medical Ethical Committee of Maastricht University.

### Data analysis

We conducted an exploratory factor analysis to explore the construct and underlying factor structure of the questionnaire. We aimed at an integrated approach in analyzing the construct of the overall questionnaire and were interested in potential overlap in the domains of the two parts of the questionnaire. Analyses were performed using principal axis factoring. Oblique rotation (Promax) was used to simplify and clarify the data structure, allowing for correlation between factors. Initially, factors with an eigenvalue of ≥1 were considered to obtain a first impression of the factor structure. In addition, inflexions in the scree plot were analyzed to estimate the possible number of factors to retain. And finally, parallel analysis was conducted to compare eigenvalues of the exploratory factor analysis with eigenvalues of a random dataset. A sample of 50 random datasets was created, using principal axis factoring with the same numbers of observations and variables as in the exploratory factor analysis. A correlation matrix was computed and eigenvalues were compared. When eigenvalues of the random datasets are larger than the eigenvalues of the factor analysis, the factors are considered due to sampling error [28]. Based on the outcome of the scree plot inflexions and parallel analysis, the number of factors was retained. Factor loadings ≥0.4 were used to assign variables to factors.

Outcomes of the exploratory factor analysis were considered accurate if all of the following criteria were met: (a) Kaiser-Meyer-Olkin measure of sampling adequacy of ≥0.7, (b) Anti-image correlations of all individual variables of >0.5, (c) Bartlett’s Test of Sphericity with a *p*-value of <0.001, and (d) a communalities average of ≥0.6 [29].

Psychometric properties of the questionnaire were analyzed to estimate internal consistency, by calculating Cronbach’s alpha of the overall questionnaire and the identified subscales. Adequate alpha values should be higher than 0.70 and values higher than 0.80 are considered excellent [30].

Barriers and facilitators for the implementation of guideline recommendations and measurement instruments were calculated using descriptive statistics. Missing values were not considered in the analysis. SAS (version 9.2) was used for statistical analysis.

## Results

### Flow and characteristics of participants

Response to the questionnaire was 57% (n = 139). Table 2 shows the distribution of responses over the regions. Average age of the participating physical therapists was 41.7 years (SD: 11.4) and 50% was male. Average years of working experience was 16.3 (SD: 10.1) years, while specific experience in treating patients with COPD was 7.5 years (SD: 6.8). Most participants (86%) worked in a primary health care setting, while the remainder (14%) worked in hospitals or rehabilitation centres.

### Exploratory factor analysis

The eigenvalues of twelve factors were >1 and the scree plot showed an inflexion at 4 factors. Parallel analysis resulted in 5 factors with higher eigenvalues in the random dataset than the eigenvalues in the exploratory factor analysis. Based on the scree plot and parallel analysis, both 4-factor and 5-factor solutions were considered and the results are presented in Table 3. The number of variables loading on each factor ranged from 5–14 in the 4-factor solution, while the range in the 5-factor solution was 2–14. Explained variance of the 4-factor solution was 36% and the 5-factor-solution explained 39% of the variance. All criteria for accuracy were met. Factor correlations ranged from 0.12-0.29 for the 4-factor solution and from −0.16-0.30 for the 5-factor solution. We characterized the content of the underlying domains of the 5-factor solution as: attitude towards using measurement instruments, knowledge and skills of the physical therapist, applicability of the COPD guideline, required investment of time & money, and patient characteristics.

**Table 3:** Exploratory factor analysis of overall questionnaire (n = 46 items)

**Factors**	**Domains**	Items*	Alpha**	Var***
4	Attitude for using MI	25,26,28,29,30,31,32,33,34,35,36,37,39,41	0.92	36%
	Knowledge and skills PT	5,6,7,20,21,38,40,42	0.80	
	Applicability of guideline	8,9,10,13,15,17,18	0.77	
	Time & Money/Patients	14,19,23,24,45	0.66	
5	Attitude for using MI	25,26,28,29,30,31,32,33,34,35,36,37,39,41	0.92	39%
	Knowledge and skills PT	3,5,6,7,20,21,38,40,42	0.76	
	Applicability of guideline	8,9,10,13,15,17,18,28	0.79	
	Time & Money	14,16,44,45,46	0.71	
	Patients	23,24	0.76	

Using principal axis factoring with oblique (Promax) rotation. Kaiser-Meyer-Olkin measure of sampling adequacy was 0.76 and p-value for Bartlett’s Test of Sphericity was <0.001 for all analyses. For all analyses individual results for Anti-image correlations were >0.5; MI: Measurement Instruments; PT: Physical Therapist.

*Variables presented with loadings ≥ 0.4; **Cronbach’s alpha;***Explained variance by factors combined.

### Psychometric properties

Cronbach’s alpha of the overall questionnaire was 0.90. In the 4-factor solution, alpha’s ranged from 0.66 to 0.92 for the different factors. The 5-factor solutions showed alpha’s ranging from 0.74 to 0.92 (Table 3).

### Barriers and facilitators

Main barriers and facilitators for implementing the COPD guideline and measurement instruments are summarized in Table 4. We combined the responses ‘strongly disagree’ and ‘disagree’ as well as ‘strongly agree’ and ‘agree’, thus resulting in three response categories. The main barriers are related to time and money. Working according to the guideline would require a higher fee for service (41%), and it takes too much time to pretest (40%) and work with the COPD guideline (37%). Main facilitators are that the recommendations in the COPD guideline allow for individual decision making (83%), and for including patient preferences (80%). The participating physical therapists responded having enough skills (81%) and knowledge (80%) to apply the COPD guideline. Responses to questions about collaboration with other health care practitioners showed that the participating physical therapists felt more supported by chest physicians (43%) than general practitioners (28%) in adopting the COPD guideline.

**Table 4:** Main barriers and facilitators for implementing the COPD guideline and measurement instruments

**Barriers**	**(Strongly) Disagree**	**Neither agree nor disagree**	**(Strongly) Agree**
Working with the COPD guideline requires a higher fee for service	27%	32%	41%
I cannot try elements to adopt the COPD guideline without much time investment^a^	37%	22%	40%
Working with the guideline COPD takes too much time.	35%	27%	37%
General practitioners do not collaborate in adopting the COPD guideline	27%	46%	27%
**Facilitators**			
MI support me in clinical reasoning and decision making	4%	6%	91%
MI provide information beyond my own professional views	4%	5%	91%
MI support my diagnostic process	4%	6%	90%
The COPD guideline allows me to make my own decisions	7%	10%	83%
I have skills to apply the COPD guideline^a^	8%	11%	81%
I have knowledge to apply the COPD guideline^a^	10%	10%	80%
The COPD guideline allows me to include patient preferences	7%	13%	80%

^a^Positive/negative phrasing of these items have been reversed for uniform presentation.

MI: Measurement Instruments.

The chest physical therapists showed a positive attitude towards measurement instruments. Physical therapists (strongly) agreed with statements that measurement instruments support the diagnostic process (90%), provide additional information to clinical expertise (91%), and are important for clinical reasoning and clinical decision making (91%). Accessibility to measurement instruments (96%) is an important facilitator for using them. Full listing of the responses to each item of the questionnaire and distribution among all response categories is presented in Additional file 1.

## Discussion

This study showed that the construct of the developed questionnaire revealed relevant underlying domains for the identification of barriers and facilitators for implementing the COPD guideline and its recommended measurement instruments within the target group of physical therapists. A practical approach was useful in constructing an overall questionnaire based on previously developed questionnaires. The factor analysis allowed for exploring the content and meaning of the underlying domains, as well as the relevance of the questionnaire to develop implementation strategies.

### Construct of the questionnaire

The characteristics of the identified factors in the 5-factor solution reflect the domains guideline (applicability of the COPD guideline), provider (knowledge & skills; attitude), context (required investment of time & money), and patient (patient characteristics) from the Peters questionnaire [21]. However, the 5-factor solution resulted in only two variables loading on the fifth factor (patients), while it is recommended that at least three variables should be represented in each common factor [28]. In the 4-factor solution the domains time & money and patients were combined with four variables, although this factor showed an insufficient alpha for internal consistency [30]. The small sample size of our study does not allow for clear conclusions about the best factor solution, but we considered the 5-factor solution most relevant. Patient characteristics are considered important in addressing barriers to implementation [13,14]. More data are needed to determine the robustness of the identified factors.

Our analysis showed little overlap of items of the two parts of the questionnaires across the identified domains, supporting the presentation of the questionnaire in two parts. However, we advocate an integrated approach in the further use of the questionnaire. Measurement instruments are core elements in guideline recommendations, and specific barriers towards the use of these instruments are important to address.

### Relevance of the questionnaire

The identified underlying domains in the questionnaire are considered important for implementation of clinical guidelines, and reflect existing frameworks for barriers to adhering to guideline recommendations [10,14]. However, the question remains whether the content of the questionnaire is actually valid for designing an implementation strategy for the COPD guideline. The main barriers for implementation were required time investment and financial constraints, which have also been reported as barriers in comparable studies in other professions [31,32]. These barriers may be considered generic and the questionnaire does not provide specific information for addressing these barriers for the specific purpose of implementing the COPD guideline.

The main facilitators show that participating physical therapists were very positive about using the COPD guideline and its recommended measurement instruments and reported to have enough knowledge and skills. Outcomes of studies that assessed barriers and facilitators for using guidelines and measurement instruments in the field of physical therapy vary, and negative [8] as well as positive attitudes [23,24] have been reported. One study reported lack of knowledge as important barrier among general physical therapists [24], as opposed to another study in which specialized physical therapists reported to have sufficient knowledge [23]. The participation of physical therapists in specialized COPD networks in our study may have contributed to the positive attitude and focus on the COPD guideline to support clinical decision-making.

Despite the positive attitude of the participating physical therapists towards using the COPD guideline, we also noted barriers to working with the guideline especially related to external factors (resources, collaboration with general practitioners). Several factors may contribute to these barriers. One aspect is that organizational and external prerequisites may be lacking, thus limiting the feasibility for adhering to certain guideline recommendations. But these barriers may also be related to lack of specific competencies and routine of physical therapists. This requires more in-depth analysis based on the results of the questionnaire and can consequently be addressed in implementation planning. The complexity of COPD care requires a multidisciplinary approach, for which quality improvement collaboratives may be of added value to address shared barriers with other disciplines [33].

Although the results of the questionnaire provide a good start for further implementing the guideline, more specific information is required for designing an implementation strategy. The lack of specific and targeted information from generic questionnaires has also been reported in other professions. A commonly used questionnaire in nursing to assess barriers to utilization of research is the BARRIERS scale. Although the scale is reliable in its use among nurses, the validity of the scale has been questioned due to its nonspecific nature and lack of evidence in being useful for designing implementation interventions [34]. Lugtenberg [22] conducted a survey among Dutch general practitioners to identify barriers for adhering to four different guidelines. The survey was derived from the results of a qualitative focus group study and specifically tailored to key recommendations in guidelines, rather than on barriers to guidelines as a whole. Their study resulted in a large variety of perceived barriers across recommendations, revealing more detailed information on potential interventions needed to improve guideline adherence [22,35].

### Methodological considerations

While the full development of new measurement scales requires a comprehensive approach [25,26], we were able to use a practical approach in constructing the questionnaire and consequently assess the underlying factor structure and relevance of the questionnaire. The main rationale was the availability of the comprehensively constructed questionnaire of Peters among several groups of health providers in the Netherlands [21], and the availability of a questionnaire that was directly applicable to the target group of physical therapists [24].

In conducting the exploratory factor analysis, several decisions were related to choosing the appropriate method for extracting factors. We chose the method of principal axis factoring with oblique rotation. Principal axis factoring allowed for comparing results with parallel analysis for the number of actors to retain, and oblique rotation was used based on the assumption that barriers for implementation in the different domains were correlated. To estimate consistency of the results, we repeated the analyses using the alternative methods of maximum likelihood and orthogonal rotation [36]. The results of these alternative analyses showed similar factor solution with some small differences in the loading of items on the different factors. The outcomes of the alternative analyses are available via the corresponding author.

Main limitation of this study for interpreting the results of the factor analysis is the relatively low sample size of 139 participants, which may have compromised the robustness of the analysis. It is recommended to use a subject to item ratio of at least 5:1 while a 10:1 ratio is considered as rule of thumb for determining a priori sample size [36,37], even though a survey among 303 published studies showed that more than 40% used a ratio of <5:1 [37]. The ratio in our study was 3:1 and as a result we need to be cautious when interpreting the outcomes, although alternative analysis showed stable results. Additional analysis of the questionnaire with a larger sample would benefit confirming the underlying factor structure. The response rate of 57% contributed to the small sample size. We did not conduct a non-response analysis and therefore have no specific information about the non-responders. Several factors may have contributed to non-responses. We used only one method for administration of the survey and people may not have felt comfortable with the online form management system. Physical therapists with a positive attitude towards guidelines may have been more inclined to fill out the survey, which could have biased our findings, in overestimating the positive attitude towards the COPD guideline [25,26].

### Implications for further strategy

The importance of tailoring interventions to prospectively identified barriers provides a clear rationale for the further development of methods to identify barriers [20]. However, the actual contribution of barrier analysis for developing implementation strategies is still considered a black box [38]. Quantitative assessment of barriers via surveys and qualitative methods via in-depth interviews are often combined and allow for in-depth analysis. Specific tailoring of surveys to key recommendations in guidelines may result in better insight in specific barriers at different levels and reveal more detailed information to develop implementation interventions [22,35]. These barriers should then be integrated in methods for designing interventions such as intervention mapping [16] or the behavior change wheel approach [19].

The construct of the developed questionnaire revealed relevant underlying domains for the identification of barriers and facilitators for implementing the COPD guideline and its recommended measurement instruments within the target group of physical therapists. The domains need to be confirmed in a follow-up study with a large sample size. While the positive attitude of physical therapists towards using the COPD clinical guideline is promising for further implementation, the results of the questionnaire alone do not provide sufficient information to inform the development of an implementation strategy.

Focus on the further development of methods for prospective identification of barriers and consequent tailoring of implementation interventions is required. The questionnaire allows for tailoring to the target group and may be used across health care professionals as basis for in-depth analysis of barriers to specific recommendations in guidelines. Efficiency and effectiveness can be enhanced by identifying specific barriers during the guideline development process. The infrastructure set up for developing the guideline can then be used for addressing key barriers by the guideline development group, using the questionnaire as well as in-depth analysis e.g. via focus group interviews. This would result in a (draft) strategy upon completion of the guideline allowing for swift progress after publication and dissemination. In addition, the identification of specific barriers during the development process will inform the guideline development group to modify the content of guidelines and to increase their usability [39].

## Conclusions

The construct of the questionnaire revealed relevant underlying domains for the identification of barriers and facilitators for implementing the COPD guideline. The questionnaire allowed for tailoring to the target group and may be used across health care professionals as basis for in-depth analysis of barriers to specific recommendations in guidelines. The results of the questionnaire alone do not provide sufficient information to inform the development of an implementation strategy. The infrastructure for developing the guideline can be used for addressing key barriers by the guideline development group, using the questionnaire as well as in-depth analysis such as focus group interviews. Further development of methods for prospective identification of barriers and consequent tailoring of implementation interventions is required.

## Competing interests

The authors declare that they have no competing interests.

## Authors’ contributions

PW carried out the planning, data analysis and writing of the manuscript. CZ contributed to the planning and data analysis of the manuscript. SD participated in the data analysis and writing of the manuscript. EH contributed to statistical analysis of the manuscript. MN participated in writing of the manuscript. RB contributed to planning and writing of the manuscript. All authors read and approved the final manuscript.

## Pre-publication history

The pre-publication history for this paper can be accessed here:

http://www.biomedcentral.com/1472-6963/13/159/prepub

## Supplementary Material

Additional file 1 Full questionnaire and detailed responses for identifying barriers and facilitators for implementing the COPD Clinical Practice Guideline. Click here for file
