# Supplementary material for: Developing a questionnaire to identify perceived barriers for implementing the Dutch physical therapy COPD clinical practice guideline
Source: BMC Health Serv Res. 2013 May 1;13:159. doi: 10.1186/1472-6963-13-159 (PMC3645972; doi:10.1186/1472-6963-13-159)
Supplement: Additional file 1 — Full questionnaire and detailed responses for identifying barriers and facilitators for implementing the COPD Clinical Practice Guideline. [file 1472-6963-13-159-S1.docx]

**Additional file**

**Full questionnaire and detailed responses for identifying barriers and facilitators for implementing the COPD Clinical Practice Guideline**

**Part 1: Barriers and facilitators for implementing the COPD guideline**

| # | Item | Strongly  Disagree  N (%) | Disagree  N (%) | Neither Agree nor disagree  N (%) | Agree  N (%) | Strongly  Agree  N (%) |
| --- | --- | --- | --- | --- | --- | --- |
| 1 | The COPD CPG allows me to make my own decisions | 1 (0,7) | 8 (5,8) | 14 (10,1) | 110 (79,1) | 6 (4,3) |
| 2 | Using the COPD CPG allows me to include patient preferences | 1 (0,7) | 9 (6,5) | 18 (13,0) | 104 (75,4) | 6 (4,3) |
| 3 | The COPD CPG provides a good basis for my self-education | 3 (2,2) | 7 (5,0) | 30 (21,6) | 93 (66,9) | 6 (4,3) |
| 4 | The COPD CPG can be misused for medical penalty law* | 4 (2,9) | 32 (23,4) | 95 (69,3) | 3 (2,2) | 3 (2,2) |
| 5 | I did not study details of recommendations in the COPD CPG* | 13 (9,4) | 71 (51,5) | 30 (21,6) | 24 (17,3) | 1 (0,7) |
| 6 | I would like to know more about the COPD CPG before I decide to adopt* | 15 (10,9) | 78 (56,5) | 28 (20,3) | 14 (10,1) | 3 (2,2) |
| 7 | I have difficulty changing my old routines* | 16 (11,5) | 71 (51,1) | 26 (18,7) | 24 (17,3) | 2 (1,4) |
| 8 | I think that certain elements of the COPD CPG are not correct* | 8 (5,8) | 68 (49,3) | 49 (35,5) | 12 (8,7) | 1 (0,7) |
| 9 | In general I do not prefer to use protocols* | 23 (16,7) | 72 (52,5) | 18 (13,0) | 22 (15,9) | 3 (2,2) |
| 10 | My colleagues do not collaborate in adopting the COPD CPG* | 21 (15,1) | 71 (51,5) | 30 (21,6) | 16 (11,5) | 1 (0,7) |
| 11 | General Practitioners do not collaborate in adopting the COPD CPG* | 4 (2,9) | 34 (24,5) | 64 (46,0) | 33 (23,7) | 4(2,9) |
| 12 | Managers do not collaborate in adopting the COPD CPG* | 19 (13,8) | 60 (43,5) | 49 (35,5) | 7(5,1) | 3 (2,2) |
| 13 | Patients do not collaborate in adopting the COPD CPG* | 17 (12,2) | 78 (56,1) | 30 (21,6) | 13 (9,4) | 1 (0,7) |
| 14 | Working with the COPD CPG takes too much time* | 3 (2,2) | 46 (33,1) | 38 (27,3) | 47 (33,8) | 5 (3,6) |
| 15 | Working with the COPD CPG clashes with my own professional practice* | 11 (8,0) | 90 (65,2) | 24 (17,4) | 12 (8,7) | 1 (0,7) |
| 16 | Working with the COPD CPG requires a higher fee for service* | 2 (1,4) | 35 (25,4) | 44 (31,9) | 48 (34,8) | 9 (6,5) |
| 17 | The lay-out of the COPD CPG is easy to use in daily practice | 3 (2,2) | 22 (15,8) | 49 (35,3) | 64 (46,0) | 1 (0,7) |
| 18 | Recommendations in the COPD CPG are clear and understandable to me | 2 (1,4) | 6 (4,3) | 30 (21,6) | 97 (69,8) | 4 (2,9) |
| 19 | I can try elements to adopt the COPD CPG without much time investment | 4 (2,9) | 52 (37,4) | 31 (22,3) | 51 (36,7) | 1 (0,7) |
| 20 | I lack knowledge to apply the COPD CPG* | 16 (11,5) | 94 (68,1) | 14 (10,1) | 14 (10,1) | 0 (0,0) |
| 21 | I lack skills to apply the COPD CPG * | 15 (10,8) | 97 (69,8) | 15 (10,8) | 12 (8,6) | 0 (0,0) |
| 22 | Chest physicians support using the COPD CPG | 1 (0,7) | 12 (8,7) | 66 (47,8) | 54 (39,1) | 5 (3,6) |
| 23 | The COPD CPS is applicable to patients with low social economic status (SES) | 6 (4,3) | 21 (15,1) | 44 (31,7) | 68 (48,9) | 0 (0,0) |
| 24 | The COPD CPG is applicable to patients with different cultural backgrounds | 1 (0,7) | 25 (18,1) | 61 (44,2) | 51 (37,0) | 0 (0,0) |

CPG: Clinical Practice Guideline

*Original formulation of items and responses are presented. For data analysis the responses of these negatively formulated items were reversed.

**Part 2: Barriers and facilitators for using measurement instruments**

| # | Item | Strongly disagree  N (%) | Disagree  N (%) | Neither Agree nor disagree  N (%) | Agree  N (%) | Strongly Agree  N (%) |
| --- | --- | --- | --- | --- | --- | --- |
| 25 | Using MI provides information beyond my professional views | 1 (0,7) | 4 (2,9) | 7 (5,0) | 97 (69,8) | 29 (21,0) |
| 26 | Using MI should be mandatory | 3 (2,2) | 14 (10,1) | 34 (24,5) | 74 (53,2) | 14 (10,1) |
| 27 | Physical therapists should only use reliable and valid MI | 0 (0,0) | 16 (11,5) | 22 (15,8) | 89 (64,0) | 12 (8,6) |
| 28 | Physical therapists are able to use MI for objective judgment of the patient’s health problem | 2 (1,4) | 10 (7,2) | 34 (24,5) | 85 (61,2) | 8 (5,8) |
| 29 | Using MI supports in clinical reasoning and responsible decision making. | 1 (0,7) | 4 (2,9) | 8 (5,8) | 105 (75,5) | 21 (15,1) |
| 30 | Using MI supports my diagnostic process | 2 (1,4) | 4 (2,9) | 8 (5,8) | 108 (77,7) | 17 (12,2) |
| 31 | Patients appreciate the use of MI | 1 (0,7) | 11 (8,0) | 32 (23,4) | 82 (59,9) | 11 (8,0) |
| 32 | Physical therapists should present treatment results using MI | 2 (1,4) | 8 (5,8) | 22 (15,9) | 93 (67,4) | 13 (9,4) |
| 33 | With objective measures I can inform patients better about their prognosis | 3 (2,2) | 13 (9,4) | 38 (27,3) | 80 (57,6) | 5 (3,6) |
| 34 | I can use objective measurements in my negotiations with stakeholders | 1 (0,7) | 2 (1,4) | 17 (12,2) | 105 (75,5) | 14 (10,1) |
| 35 | Using MI supports making a diagnosis | 1 (0,7) | 10 (7,2) | 12 (8,7) | 100 (72,5) | 15 (10,9) |
| 36 | Using MI increases the objectivity of treatment results | 2 (1,4) | 4 (2,9) | 13 (9,4) | 108 (77,7) | 12 (8,6) |
| 37 | Using MI supports my clinical reasoning | 1 (0,7) | 3 (2,2) | 13 (9,4) | 107 (77,0) | 15 (10,8) |
| 38 | I find scores of MI difficult to interpret* | 10 (7,3) | 69 (50,4) | 40 (29,2) | 18 (13,1) | 0 (0,0) |
| 39 | I do not have MI available and do not know where to find them* | 71 (51,4) | 62 (44,9) | 2 (1,4) | 2 (1,4) | 1 (0,7) |
| 40 | I cannot judge whether MI are of sufficient quality* | 18 (12,9) | 65 (46,8) | 40 (28,8) | 16 (11,5) | 0 (0,0) |
| 41 | I am unfamiliar with MI* | 49 (35,5) | 77 (55,4) | 5 (3,6) | 5 (3,6) | 3 (2,2) |
| 42 | I am insufficiently educated to use MI* | 40 (29,6) | 67 (49,6) | 14 (10,4) | 12 (8,9) | 2 (1,5) |
| 43 | Current MI are not suitable for physical therapy practice* | 33 (23,7) | 82 (59,0) | 17 (12,2) | 7 (5,0) | 0 (0,0) |
| 44 | Using MI is too expensive (buying, copies)* | 20 (14,4) | 63 (45,3) | 35 (25,2) | 18 (12,9) | 3 (2,2) |
| 45 | Using MI takes too much time* | 8 (5,8) | 62 (44,6) | 33 (23,7) | 30 (21,6) | 6 (4,3) |
| 46 | The use of MI is an unnecessary burden for patients * | 15 (10,8) | 83 (59,7) | 26 (18,7) | 13 (9,4) | 2 (1,4) |

MI: Measurement Instruments

*Original formulation of items and responses are presented. For data analysis the responses of these negatively formulated items were reversed.
